# Technical nuances of robotic-guided S2 alar-iliac screw insertion

**DOI:** 10.3171/2025.4.FOCVID255

**Published:** 2025-07-01

**Authors:** Pradeep Vitta, Abhishek Soni, Balamurugan Thirugnanam, Madhava Pai Kanhangad, Vidyadhara Srinivasa

**Affiliations:** 1 Manipal Institute of Robotic Spine Surgery, Manipal Comprehensive Spine Care Centre, Manipal Hospital, Bangalore, India; and; 2 Department of Orthopaedics, Kasturba Medical College Manipal, Manipal Academy of Higher Education, Manipal, India

**Keywords:** S2AI screw placement, innovations in robotic sacropelvic fusion, spinal robotics evolution, sacral alar fixation, robot-guided percutaneous fixation

## Abstract

Robot-assisted S2-alar-iliac (S2AI) screw placement enhances accuracy and minimizes complications in spinal deformity and pelvic fixation surgeries. This video outlines the intraoperative navigation, planning, and execution of S2AI screw placement using robotic guidance. By optimizing screw trajectory and reducing soft-tissue disruption, robotic assistance improves biomechanical stability in long-segment spinopelvic constructs. The integration of real-time navigation enhances precision, potentially lowering revision rates. This video aims to highlight the technical nuances, clinical advantages, and outcomes associated with robot-assisted S2AI screw placement, underscoring its role in advancing spine surgery with greater consistency and safety.

The video can be found here: https://stream.cadmore.media/r10.3171/2025.4.FOCVID255

## Transcript

### 0:25

We will be going into the technical nuances of robot-assisted sacroiliac screws, which are used in lumbosacral fixation. These are also commonly known as S2-alar-iliac (S2AI) screws because of their entry points and are a standard of practice in long fusions in the lumbar spine.^1^

### 0:42

In this video we are using the Mazor X Stealth Edition from Medtronic, which is their third-generation robotic system, running software version 5.1.2.

### 0:53

The patient is a 47-year-old lady who had presented with complaints of pain in the lower back region radiating to the bilateral lower limbs, along with progressive difficulty in walking. She also had complaints of sensory dysesthesias in the lower limbs along the L5–S1 dermatomal distribution. There was history of progressive neurogenic claudication. There were no complaints of altered bowel or bladder habits. On clinical examination, the bulk and tone were normal. Power was decreased in the lower limbs, with grade 4 across all muscle groups in the bilateral lower limbs. Sensory examination revealed a mild impairment to perception of light touch in the bilateral L5–S1 dermatomes. DTRs were decreased across the bilateral knee and ankle joints.

### 1:41

X-ray of the lumbosacral spine in AP and lateral views showed a degenerative scoliosis of the lumbar spine with convexity to the right and degenerative changes of the lumbosacral spine, while an MRI showed multilevel disc prolapses and a grade 1 listhesis at the L3–4 and L4–5 levels with varying degrees of foraminal stenosis in the lumbar spine.

### 2:04

The patient was positioned prone on an Allen Advance Table with the arms abducted at the shoulder to 90° and placed in padded arm supports alongside the head.

### 2:14

The placement of S2AI screws involves minor modifications in the workflow compared to thoracolumbar pedicle screw placement with robot assistance. Broadly, the steps are the usual "scan and plan" workflow with the field-of-view set to 40 cm on the O-arm, followed by planning of screws in the ilium, verification of the trajectory using navigated instruments and planning software, positioning the robotic arm to the specified trajectory, and, finally, insertion of the S2AI screws.

### 2:47

Schanz’s pin is inserted into the ilium, ensuring it does not interfere with the planned S2AI screws. It is checked for a sturdy hold, and the robot is physically mounted onto the patient using this Schanz’s pin with the help of a bridge.

### 3:02

The region of interest is then covered with a nonreflective sheet. A 3Define scan is performed to identify possible obstacles in the path of the robotic arm. While the 3Define scan is going on, the scrub nurse verifies the instruments by placing them over the reference frame divot. A snapshot of the robotic arm is then taken to register the navigation frame position relative to the arm. Region of interest is marked using a blunt passive planar probe, and the robotic arm is sent to the marked position. A star marker is attached to the robot’s arm and positioned as close to the patient’s body as is possible to cover more segments.

### 3:41

The O-arm is then brought in to perform an intraoperative CT with the patient fully covered by a disposable plastic drape.

### 3:49

All four beads of the star marker must be clearly visualized on the 3D scan. This can be ensured by taking orthogonal x-rays of the surgical area before the final spin. The key to covering a larger area is positioning the star marker close to the skin.

### 4:05

During the O-arm scan, a 20-cm field-of-view, or FOV, is standard and is typically used for placing cervical and thoracolumbar pedicle screws. A 40-cm FOV is used for S2AI screw insertion and iliosacral screw placement. It is crucial to ensure that both iliac wings are fully visible when acquiring a 40-cm FOV scan.

### 4:28

After the O-arm scan, planning begins by marking the region of interest, roughly outlining the spinal canal in the area. The robot then provides probable segmentation, which is verified and labeled with reference to the sacral vertebrae. Once segmentation is complete, planning for the iliac screws starts. The screws are initially oriented roughly along the ilium in the sagittal, coronal, and axial planes. Adjustments are made while scrolling through the CT cuts, ensuring the screw trajectory avoids any breach in the bony cortices. Specific attention is given to the sciatic notch, iliac tables, and acetabulum.

### 5:07

After planning the trajectory of the screws, the incision is checked using the software’s skin-level feature, aiming to place both S2AI screws through a single midline incision. Once planning is finalized the process proceeds to screw insertion.

### 5:22

A common error at this stage is setting the region of interest too narrow on the robot console. If this happens, even if a wider image is acquired the robot will only display the cropped image. During the planning phase, attention must be paid to the axial, coronal, and sagittal sections. In the coronal view, the S2AI screw should start at the midpoint between the S1 and S2 foramina, lateral to the foraminal border. The screw should be planned with 40°–50° of lateral angulation and 20°–30° of caudal angulation, with a minimum length of 80 mm.^2,3^ The angle of entry at the iliac surface of the SI joint influences the risk of skiving, with more obtuse angles posing a greater risk.

### 6:07

This clip shows the insertion of the left iliac screw. For percutaneous S2AI screw insertion, the skin is first incised twice in opposite directions, so as to comfortably fit the robotic instruments without being subjected to traction by soft tissue, fascia, or the muscles. After positioning the robotic arm to the planned trajectory, a sleeve with a trocar is then placed, and once it sits on the bone, a pilot hole is created using a high-speed drill. Since the pilot hole may not cross the SI joint, a 4-mm tap is passed to extend it beyond the joint. This is followed by tapping with a 6.5-mm tap. And finally, the iliac screw is inserted to the desired depth under navigation guidance. The same steps are followed for inserting the right-side screw. This workflow allows surgeons to place S2AI screws percutaneously, minimizing the need for extensive exposure and blood loss. Additionally, it eliminates extra radiation exposure to surgeons, staff, and patients, unlike fluoroscopy-guided sacroiliac screws.^4^

### 7:12

The nonavailability of a longer 3-mm high-speed drill bit is a challenge that needs to be addressed. Our workflow was designed using the available instruments. The 4.0-mm awl-tipped tap is sharp, which helps prevent skiving and allows it to cross the SI joint with a lower risk of maltracking. In most cases, the planning closely matches the execution.

### 7:33

Let’s reiterate the steps for S2AI screw fixation. Drill a pilot hole using a 3.0-mm drill bit through the sleeve and arm guide. Tap with the 4.0-mm awl-tipped tap, which is a critical step for maintaining the trajectory to a depth of 60 mm. Tap with the 6.5-mm tap to ensure the entire length of the trajectory is prepared. Insert the 7.5- or 8.5-mm S2AI screw using power tools to minimize maltracking. Verify the final position using fluoroscopy.

### 8:05

One of our patient’s postoperative CT is shown here, demonstrating perfect placement of S2AI screws with no breaches. Postoperative 3D CT shows proper placement of implants. The robot also facilitates the planning and placement of S1-alar-iliac screws, which can be used alongside S2-iliac screws for enhanced spinopelvic stabilization. The starting point for the S1-alar-iliac screws is just lateral to the junction of the S-1 superior articular facet and the posterior sacral ala, which is in proximity to the entry point for S-1 pedicle screws. It is 3–5 mm above the superolateral corner of the S-1 foramen. The double screws may help in achieving greater spinopelvic stability.^5^

### 8:49

Few advantages of S2AI iliac screws over iliac screws or bolts are that it is associated with lesser risk of complications such as screw prominence, gluteal pain, and wound breakdown, primarily because of iliac bolts’ superficial PSIS entry point.^6^

### 9:04

Compared with freehand insertion, robotic insertion of S2AI screws has multiple advantages such as avoidance of screw misplacement, breach of cortical bone, and injury to neurovascular structures, which are more often seen in manual insertion.

### 9:21

While opting for the "scan and plan" workflow on Mazor X system, there is no need of a preoperative CT scan, as this additional scan would expose the patient to unnecessary radiation and there might be a discordance between the intraoperative positioning and the positioning obtained during the preoperatiave CT imaging. For these reasons we opt for an intraop-obtained "scan and plan" workflow.

### 9:44

Another important point to note is that while using the drills, one has to drill intermittently while appreciating the haptic feedback at the tip of the screw every time before restarting. This can help in minimizing lateral breach.

## Disclosures

The authors report no conflict of interest concerning the materials or methods used in this study or the findings specified in this publication.

## Author Contributions

Primary surgeon: Srinivasa. Assistant surgeon: Vitta, Soni, Thirugnanam, Kanhangad. Editing and drafting the video and abstract: Vitta, Soni. Critically revising the work: Vitta, Soni, Thirugnanam. Reviewed submitted version of the work: Srinivasa, Vitta, Soni, Thirugnanam. Approved the final version of the work on behalf of all authors: Srinivasa. Supervision: Srinivasa, Vitta.

## Supplemental Information

### Patient Informed Consent

The necessary patient informed consent was obtained in this study.

## References

[1] Park PJ Lin JD Makhni MC Cerpa M Lehman RA Lenke LG Dual S2 alar-iliac screw technique with a multirod construct across the lumbosacral junction: obtaining adequate stability at the lumbosacral junction in spinal deformity surgery Neurospine 2020 17 2 466 470 31694359 10.14245/ns.1938320.160 PMC7338953

[2] Shillingford JN Laratta JL Park PJ Human versus robot: a propensity-matched analysis of the accuracy of free hand: versus: robotic guidance for placement of S2 alar-iliac (S2AI) screws Spine (Phila Pa 1976) 2018 43 21 E1297 E1304 29672421 10.1097/BRS.0000000000002694

[3] DePasse JM Valdes M Palumbo MA Daniels AH Eberson CP S-1 alar/iliac screw technique for spinopelvic fixation J Neurosurg Spine 2018 28 5 543 547 29393830 10.3171/2017.8.SPINE16904

[4] Arora A Berven S Challenges and complications in freehand S2-alar-iliac spinopelvic fixation and the potential for robotics to enhance patient safety Global Spine J 2022 12 2_suppl 45S 52S 35393876 10.1177/21925682211036664 PMC8998478

[5] Mattei TA Fassett DR Combined S-1 and S-2 sacral alar-iliac screws as a salvage technique for pelvic fixation after pseudarthrosis and lumbosacropelvic instability J Neurosurg Spine 2013 19 3 321 330 23808582 10.3171/2013.5.SPINE121118

[6] Ishida W Elder BD Holmes C S2-alar-iliac screws are associated with lower rate of symptomatic screw prominence than iliac screws: radiographic analysis of minimal distance from screw head to skin World Neurosurg 2016 93 253 260 27319308 10.1016/j.wneu.2016.06.042

